# Membranes of Polymers of Intrinsic Microporosity (PIM-1) Modified by Poly(ethylene glycol)

**DOI:** 10.3390/membranes7020028

**Published:** 2017-06-05

**Authors:** Gisela Bengtson, Silvio Neumann, Volkan Filiz

**Affiliations:** Helmholtz-Zentrum Geesthacht, Institute of Polymer Research, Max-Planck-Strasse 1, 21502 Geesthacht, Germany; gisela.bengtson@hzg.de (G.B.); silvio.neumann@hzg.de (S.N.)

**Keywords:** polymers of intrinsic microporosity (PIM-1), poly(ethylene glycol), copolymers, gas separation, membranes

## Abstract

Until now, the leading polymer of intrinsic microporosity PIM-1 has become quite famous for its high membrane permeability for many gases in gas separation, linked, however, to a rather moderate selectivity. The combination with the hydrophilic and low permeable poly(ethylene glycol) (PEG) and poly(ethylene oxides) (PEO) should on the one hand reduce permeability, while on the other hand enhance selectivity, especially for the polar gas CO_2_ by improving the hydrophilicity of the membranes. Four different paths to combine PIM-1 with PEG or poly(ethylene oxide) and poly(propylene oxide) (PPO) were studied: physically blending, quenching of polycondensation, synthesis of multiblock copolymers and synthesis of copolymers with PEO/PPO side chain. Blends and new, chemically linked polymers were successfully formed into free standing dense membranes and measured in single gas permeation of N_2_, O_2_, CO_2_ and CH_4_ by time lag method. As expected, permeability was lowered by any substantial addition of PEG/PEO/PPO regardless the manufacturing process and proportionally to the added amount. About 6 to 7 wt % of PEG/PEO/PPO added to PIM-1 halved permeability compared to PIM-1 membrane prepared under similar conditions. Consequently, selectivity from single gas measurements increased up to values of about 30 for CO_2_/N_2_ gas pair, a maximum of 18 for CO_2_/CH_4_ and 3.5 for O_2_/N_2_.

## 1. Introduction

Polymers of intrinsic microporosity have been and continue to be the objects of many research papers (see, e.g., citations in [1]) since Budd and McKeown launched their first publication about PIM-1 in 2004 [2].

PIM-1 is a hydrophobic polymer formed as a molecular ladder; the polymer chains cannot pack tightly because of their contorted molecular structure, provided by spiro fused rings in the polymer chain. Thus, intrinsic nanopores are formed resulting in a large inner surface [3]. Membranes prepared from this polymer show a large fractional free volume and therefore high permeability for many gases [4], but especially for CO_2_ (4000 to 10,000 Barrer) because of an also very high solubility [5]. However, the trade-off between permeability and selectivity, as it was evaluated by Robeson [6], consequently results for PIM-1 in a moderate permselectivity of CO_2_ over N_2_ of about 16 to 20 and this is not at all comparable to the selectivity values obtained with membranes made from polymers such as PEBAX^®^ (Arkema, Colombes, France) or Polyactive™ (Polyvation, Groningen, The Netherlands), ranging from 30 to 40 [7]. Both latter polymers are block copolymers, containing poly(ethylene (or higher equivalents) oxide) (PEO) chains for soft blocks, thus performing the transport of CO_2_ [8]. As a drawback, they are of low overall permeability for gases (e.g., 100 to 300 Barrer CO_2_). The hard blocks (polyamides) necessary for mechanical stability do not contribute to permeability at all and PEOs in general show low permeability [9]. The PEO blocks in PEBAX^®^ and Polyactive^™^ derive of ethylene or butylene glycol units and they are rather short to avoid crystallization of PEO because crystallites are almost impermeable to any gases and would additionally reduce the overall permeability [10].

In combining PIM-1 and low molecular PEG- or PEO-blocks, it is expected to get a reduced permeability but an enhanced selectivity for CO_2_ due to its preferred solubility in PEGs and also due to a reduction of permeability of non-polar gases like N_2_. In recently published research Wu et al. [11] followed this idea by blending PIM-1 and PEGs of different molecular weights (2 up to 20 kg/mol) in concentrations up to 5 wt % PEGs to enhance the selectivity of CO_2_ over N_2_ as well as over CH_4_, clearly without crucifying permeability too much. They observed a reduction to about half of the permeability of pure PIM-1 blended with 2.5 wt % PEG. With increasing molecular weight of PEG the permeability increased somewhat again coupled to a slight decrease in selectivity. Therefore, they chose PEG with 20 kg/mol for further experiments regarding PEG content and feed pressure. At 3.5 wt % PEG (20 kg/mol), a permeability of almost 2000 Barrer CO_2_ and a selectivity for CO_2_/CH_4_ of 39 was achieved. Selectivity of CO_2_ over N_2_ was highest with 2.5 wt % PEG (2 kg/mol), with a value of 23.

In our study, we followed four different paths to combine PIM-1 and PEG or PEO. First, we also blended PIM-1 with PEGs of different molecular weights to evaluate the optimum amount of PEG and to check on the influence of PEG chain length. Long PEO chains are favorable for CO_2_ solubility [10] but tend to crystallize and separate from PIM-1 phase. Therefore, we favored PEGs with smaller chain lengths of ≤5 kg/mol. The permeability of blended membranes should not be reduced too much; the influence of the added PEGs on solubility of CO_2_ and other gases is going to be estimated. A similar approach was followed in [12,13] by blending PEBAX^®^ (Arkema, France) with PEGs in different amounts, although in these cases it was thought as an attempt to increase the rather low pristine permeability of the block copolymer.

Along the following Paths 2–4, we strived for a chemical bondage between PIM-1 and PEO by different substitution reactions. In Path 2, we added PEG-monomethylethers, bound by esterification to trihydroxybenzoic acid (THBA), as quenching compounds to terminate the polycondensation reaction forming PIM-1. This resulted in (di)block copolymers with rather large PIM-1 blocks fenced by small PEO end group(s). The procedure was performed with PEGs of different chain lengths.

Within Path 3, we combined small PIM-1 blocks (at least smaller blocks than in Paths 1 and 2) with different PEO-containing co-monomers (PEG di-substituted by esterification with dihydroxybenzoic acid (DHBA)) to form multiblock copolymers (PIM1-*b*-PEO). This approach increased the influence of PEO further by avoiding large parts/blocks of pure PIM-1.

Within the fourth path, PEO were built in as side chains to the PIM-1 main chain by copolymerization with PEO substituted anthracene based maleimides. The synthesis of these maleimides followed the procedure published in [14]. The roof shaped tetrahydroxy-anthracene monomers were employed in polycondensation reaction together with the PIM-1 antecessors.

The temperature is surely an important factor for permeability of polymeric structures, with permeability being the product of diffusivity and solubility of gases in polymers [15]. While diffusion increases with rising temperature because of a higher mobility of the polymer chains, solubility usually decreases. However, PEG/PEO chains tend to crystallize with increasing molecular weight and crystallites do not contribute to permeability. At elevated temperature, these crystallites will melt, influencing diffusivity and solubility favorably [10]. Therefore, we also include measurements of gas permeability at elevated temperatures to inquire the influence of melting PEO chains.

## 2. Experimental

### Methods

(1) For descriptions of Equipment used in NMR, FT-IR, SEC, TGA and DSC, see our former paper [14].
(2) Size Exclusion Chromatography (SEC) was performed in CHCl_3_ solution, detection by Refractive Index (RI) with polystyrene standards and/or Multi Angle Light Scattering (MALS) using dn/dc increment measured at different concentrations by RI detection.
(3) Details of the synthesis of monomers and polymers are given in Supplementary Materials S1.
(4) Details of the gas separation calculation are given in Supplementary Materials S2.

## 3. Results and Discussion

The aim of this study in polymeric membrane research is the combination of PIM-1, the rigid hydrophobic high flux polymer, with hydrophilic poly(ethylene glycol) (PEGs) of various polymer weights to form stable membranes of improved gas separation properties, especially for CO_2_. Low molecular PEGs are hydrophilic viscous liquids that are (in principle) quite permeable to gases because of their very good solubility, e.g., for CO_2_, but obviously they lack the required mechanical stability necessary for a useful membrane. Higher molecular PEGs (>1000 g/mol) are waxy solids, but their crystalline structure diminishes their permeability considerably [10].

In the following (Section 3.1, Section 3.2, Section 3.3 and Section 3.4), four different approaches have been evaluated to combine PIM-1 with PEGs and PEO/PPO, described schematically in Table 1. In Section 4, a summary of the different paths is given.

### 3.1. Blend Membranes (Path 1)

Our blending of PEGs in PIM-1 membranes is a similar approach that was already followed in [11] with a PEG of 20 kg/mol. In our study, however, we used PEGs of low molecular weight, 200, 1000 and 2000 g/mol; PEGs were added in 5 and 10 wt % relative to polymer mass by dissolving in PIM-1 casting solutions (solvent CHCl_3_). The solvent was evaporated slowly in a nitrogen stream during 24 h to allow a sufficient packing of polymer chains. Dense membranes of 70–100 µm thickness were formed. They are flexible and mostly transparent with exception of the blends with PEG-2000, these are slightly turbid, caused supposedly by some partial internal phase separation. However, by blending with even longer chained PEG of 20 kg/mol phase, separation was already observed at 2.5 wt % [11].

The prepared membranes were measured with single gases (N_2_, O_2_, CO_2_, and CH_4_) in a time lag apparatus [16,17] at pressure differences below 1 bar. In the course of Path 1 we decided to compare to the measured values of a freshly prepared PIM-1 membrane originating from the identical PIM-1 batch (*M*_w_ ca. 300 kg/mol, preparation at 150 °C as described in [18] procedure III) we used for all experiments of blending. To confirm that the blend membranes are (thermally) stable, TGA was conducted before and after a time lag measurement cycle at different temperatures ranging from 20 to 80 °C. Especially low molecular PEG-200 is sufficiently volatile to be squeezed out of the membrane by repeated differences in pressure. However, less than 1% of the membranes weight was lost during the first time lag cycle measured with 4 different gases and temperatures up to 80 °C. These small losses could be easily explained by removal of some residual solvent. No further losses were observed during repetitions of time lag cycle. These results are in stark contrast to the report on blends of PEBAX^®^ and PEGs of low molecular weight (<500 g/mol) losing about 30 wt % PEG during time lag measurement [13].

Additionally, we used TGA measurements up to 500 °C to estimate the actual blend composition by the observed specific decompositions/weight losses of the PIM-1-PEG blends. While PIM-1 in Argon atmosphere is stable up to at least 430 °C, decomposition of the blending PEGs starts already below 400 °C. Therefore, the weight loss up to 400 °C was taken for a measure of the actual PEG content. Deviations from calculated compositions (5 and 10 wt %) were probably caused by the water content of the “as received” PEG samples in use for blending or by solvent residues within the PIM-1 polymer.

Setup and details of gas separation measurements are given in Supplementary Materials S2. Gas separation measurements at 30 °C are presented in Table 2, together with the measured values for the pristine PIM-1 we used for blending.

As expected from the very different basic permeability of PIM-1 and PEG, the PEG-blended membranes showed a considerable reduction of permeability compared to the measured values of pristine PIM-1 membrane. The reduction depends directly on the amount of PEG added. With ca. 5 wt % of PEG added, the permeability was reduced to 30–40%, with ca. 10 wt % PEG the permeability dropped to 10–20% of the original PIM-1 value. From these results we concluded that addition of more than 10 wt % PEG is inappropriate. Permeability reduction of CO_2_ and O_2_, however, was less severe compared to N_2_ and CH_4_ and therefore improved selectivity for CO_2_/N_2_ and for O_2_/N_2_ gas pairs were calculated by division of single gas measurements (see Table 2).

**Figure 1:**
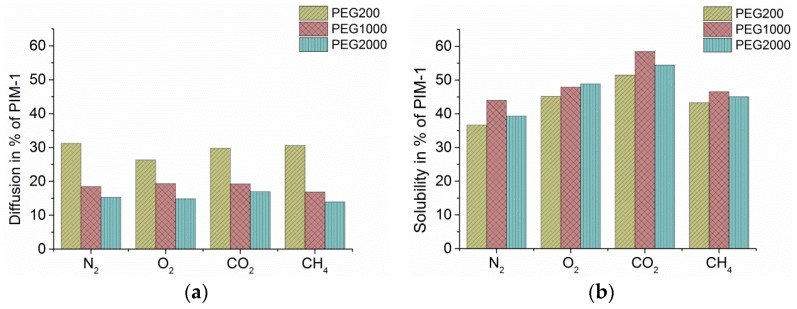
Calculated diffusivity (**a**) and solubility (**b**) of PIM-1 + PEG-blends (ca. 10 wt% PEGs) in relation to plain PIM-1 (=100%); results of time-lag experiments with single gases at 30 °C.

In Figure 1 are shown diffusivity (Figure 1a) and solubility coefficients (Figure 1b) of three blended membranes (ca. 10 wt % of the three different PEGs) calculated from time lag experiments (see Supplementary Materials S2) and set in relation to the pristine PIM-1 (=100%). As expected, the solubility of CO_2_ is reduced less compared to O_2_, N_2_, and CH_4_ (Figure 1b). It is known that enhanced solubility is the reason for the high flux of CO_2_ in PIM-1 and other polymers [19], and also in PEGs [13] while N_2_ has a low solubility in PIM-1 as well as in PEGs. Therefore, selectivity of CO_2_ over N_2_ increased up to a value of 30 at 10 wt % PEG content. The chain length of the PEGs affected solubility only slightly but with PEG-1000 in favor.

Within PIM-1, membrane diffusion of O_2_ is highest (2.41 × 10^−6^ cm^2^/s) followed by CO_2_ (0.95) and N_2_ (0.86), while CH_4_ is slowest (0.35), and introduction of PEG does not change this order. All measured gases are affected quite evenly (Figure 1a). Diffusivity is higher in blends with PEG-200 than in PEG-1000 and PEG-2000, caused probably by the absence of any crystallinity within the originally liquid PEG-200, the higher amount of the small PEG molecules and the hence better distribution of the small PEG-200 chains into the intrinsic microporosity of the PIM-1 ladders. The larger PEG molecules represent parts within the PIM-1 matrix that are of low permeability and consequently slow down diffusion of all gases.

**Figure 2:**
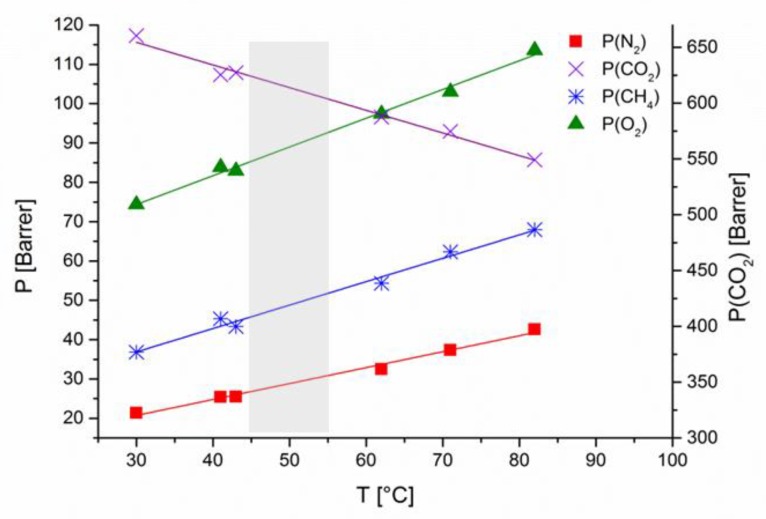
PIM-1 + 11 wt% PEG-2000: Permeability of four different gases at temperatures up to 80 °C. Colored in grey is the melting region prospected for PEG-2000.

Blends of PIM-1/PEG-2000 were measured at different temperatures from 30 to 80 °C to inquire for the influence of PEG crystallites. While PEG-200 is liquid, PEG-1000 and PEG-2000 are solids and show distinctive melting points (40 and 54 °C, respectively). PEG-2000 has the highest *M*_w_, a melting point of 53.8 °C and a crystallinity of ca. 70% was calculated from DSC (crystallinity calculated after [20]). The blend of PIM-1 + 11 wt % PEG-2000 in DSC measurement showed a (slightly) lowered melting peak at 49 °C and a very low crystallinity (<1%). It was concluded that crystallization of PEG-2000 was successfully inhibited within the PIM-1 matrix although the membrane was not fully transparent. Consequently, it was not expected to observe a deviation in thermal dependency of permeability or diffusion when compared to PIM-1. In Figure 2, permeability of four gases at temperatures from 20 to 80 °C is depicted and the prospected melting region of PEG-2000 is marked by a grey zone. Permeability of N_2_, O_2_ and CH_4_ increase linearly with temperature, while permeability of CO_2_ decreases also linearly with T; the different behaviors caused by the considerable decreasing solubility of CO_2_ within the polymer with rising temperature. As expected from measured crystallinity, the permeability showed no inconsistency in the curves depending on temperature.

**Figure 3:**
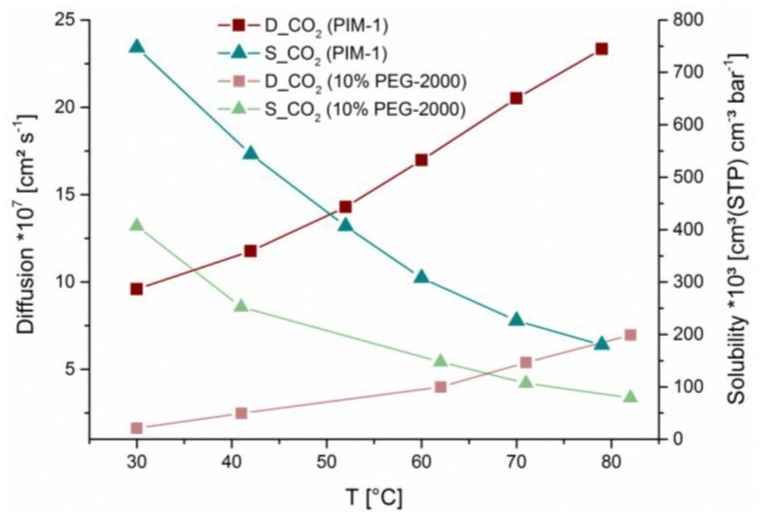
Time lag measurement of CO_2_ with PIM-1 (dark color) and PIM-1 blended with 11 wt % PEG-2000 (light color): Diffusion (left, squares) and solubility (right, triangles) of CO_2_ depending on temperature.

Because of the clearly different behavior of CO_2_ from the other measured gases, in Figure 3 diffusion and solubility of CO_2_ in pristine PIM-1 and PIM-1 blended by 11% PEG-2000 versus temperature are compared. Solubility of CO_2_ (Figure 3, left side, triangles) in blended PIM-1 membrane (pale triangles) decreased exponentially from a high level and very similar compared to PIM-1 (dark triangles), at 80 °C only 20% of the solubility coefficient at 30 °C is left. According to the low degree of crystallinity, diffusivity of CO_2_ could be described by exponential curves too (Figure 3, right side, squares). The increase in diffusivity from 30 to 80 °C is about 2.5 times for PIM-1 (dark squares) and 4 times for the blended membrane (pale squares) involving the pronounced miscibility of PIM-1 and PEG-2000 at higher temperature. The other three gases, measured but not depicted, showed a similar behavior in diffusion as well as a decrease in solubility but on a much lower level.

Gas permeation is clearly dominated by PIM-1, and the selective slow down by PEG results in better selectivity for the very soluble CO_2_ compared to N_2_ and CH_4_ (see Table 2).

At higher temperature, however, this effect is more than neutralized by the decreasing solubility of CO_2_ (Figure 3).

From these orienting results derived from blending PIM-1 and PEG, it was concluded that the amount of PEG should stay below 10 wt %, or even lower, to rescue a better part of the high permeability of PIM-1. Introduction of PEG, even one as crystalline as PEG-2000, into PIM-1 membrane does not change the temperature dependency of permeability and diffusion. Melting of PEG chains seems to have no recognizable influence on permeation behavior. Consequently, further evaluation of higher temperatures was skipped for the following paths; all following measurements were performed at 30 °C.

### 3.2. Quenching to Form PEO End Groups of PIM-1 Chains (Path 2)

**Scheme 1:**
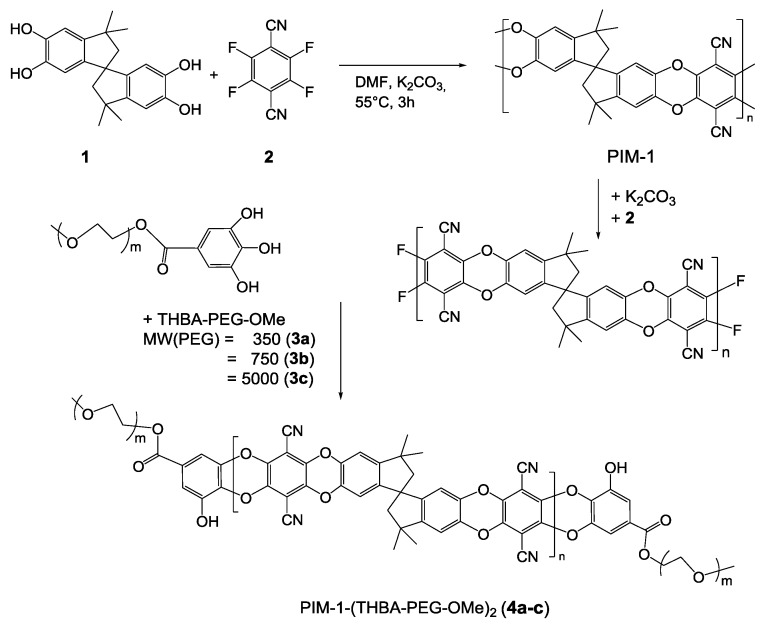
Preparation of PIM-1-(THBA-PEG-OMe)_2_ polymers (**4a–c**) by termination of PIM-1 polycondensation with THBA-PEG-OMe (**3a–c**).

Path 2 to introduce PEG and PEO into PIM-1 polymer employed the use of PEG-substituted trihydroxy-benzoic compounds to terminate the PIM-1 polycondensation reaction. Details of synthesis are given in Supplementary Materials S1. PIM-1 was prepared as described in [1] by low temperature polycondensation reaction of equimolar amounts of **1** and **2** catalyzed by potassium carbonate (see upper part of Scheme 1). The reaction runs smoothly and reproducibly in DMF at 55 °C. To achieve a film and membrane forming polymer an average *M*_w_ of about 50 kg/mol is necessary and this particular molecular weight is obtained after ca. 3 h of reaction time. Usually, the polycondensation reaction would be terminated by precipitation in water or methanol restoring the hydroxy end groups. By adding a compound, e.g., 1,2-dihydroxybenzene, as quencher, providing two aromatic hydroxyl groups, the chains’ growth would be stopped at the fluoro substituted ends. The fluoro end groups in this case are quenched preferentially by the small and therefore more easily accessible molecules of the quencher instead of PIM-1 chains. To introduce PEG structures by this method, we prepared esters of different poly(ethylene glycol) monomethyl ethers and trihydroxybenzoic acid (**3a–c**) (Scheme 1) by standard synthetic procedures and used them for quenching. THBA provides the necessary phenolic OH-groups to react as phenolate in alkaline solution with the PIM-1 fluoro ends. Within these experiments, THBA was chosen over dihydroxybenzoic acid (DHBA) to improve the availability of hydroxy groups for quenching. Poly(ethylene glycol) monomethyl ethers with molecular weights of 350 (**3a**), 750 (**3b**) and 5000 (**3c**) g/mol were employed. The latter one was included to improve the amount of PEG in comparison to a PIM-1 polymer of 50 kg/mol.

A growing PIM-1 polymer chain has fluoro as well as hydroxy end groups; therefore, a simple quenching would lead to positioning of the quenching molecule at one end of each polymer chain. To improve the amount of end groups to be quenched by THBA-PEG-OMe (**3a–c**) most end groups favorably should be fluoro substituted. Unfortunately it is not feasible to run the reaction with a considerable excess of **2** from the start, while this leads to rather short chains of low molecular weight polymer (8000 to 10,000 g/mol) without any film forming properties (compare to Section 3.3, Figure 5). Therefore, to achieve entire fluoro substitution on both ends of the polymer chains an additional small amount of **2** was added after 3 h of reaction time to react for another 15 min with the already present phenolates. The respective trihydroxy-benzoic acid-PEG-monoester **3** was added to the reaction solution, stirred for another 0.5 h and the polymer was worked up as usual by precipitation in water. The isolated polymers PIM1-(THBA-PEG-OMe)_2_ (**4a–c**) are film forming and readily soluble in CHCl_3_. They were characterized by ^1^H-NMR, FT-IR, SEC with RI/MALS (multi angle light scattering) detector, TGA and DSC (see *Polymerization example 3* in Supplementary Materials S1).

In ^1^H-NMR the signals of the PEG, –OCH_2_ groups at 3.6 ppm are clearly visible and the carboxylic group of the respective glycol esters is found at 1700 cm^−1^ in FT-IR. The molecular weights measured by SEC are quite similar to PIM-1 prepared under identical conditions and within the usually observed deviations (*M*_w_ = 48–55 kg/mol). Regarding the low molecular weights of PEG-350 and PEG-750, it was clear that the overall PEG content of polymers **4a–b** is rather low, a considerable amount was achieved only by adding PEG-5000 in polymer **4c**. In TGA measurements, the decomposition of the PEO-containing polymers **4a–c** was observed to identify the PEO-part of polymers **4a–c**. While PIM-1 is stable up to 430 °C, decomposition of PEO-containing polymers starts already below 350 °C. Therefore, it seemed reasonable to use the weight loss up to 400 °C (splitting off of PEG-esters) for a measure of the PEO containing part. Measured mass changes of the first decomposition step, re-calculated to the solely PEO content, are 0.6 (**4a**), 0.9 (**4b**) and 3.1 (**4c**) wt %. Calculation of PEG-contents via ^1^H-NMR integrals of the polymers gave similar values for **4a** of 0.5 and for **4b** of 0.9 wt %. **4c** was difficult to evaluate from ^1^H-NMR spectrum because of enclosed amounts of the quencher **3c**. After a series of washing steps, finally a content of 2.6 wt % was estimated. A probable reason for deviation from originally aspired higher content could be an only partly successful quenching; especially for the long chained PEG-5000-OMe with its restricted mobility the reaction time was probably insufficient to quench all possible fluoro ends of PIM-1.

The polymers **4a–c** were successfully formed into dense membranes by solution casting from CHCl_3_. Gas permeability of the polymers **4a–c** with four different gases at 30 °C were measured in the time lag apparatus and presented in Table 3 in comparison to a freshly prepared PIM-1 membrane of comparable molecular weight (50 kg/mol). As expected by the low contents, the influence of the PEO end groups on gas permeation performance is quite small, especially for the low molecular PEGs added in **4a** and **4b**. The gas permeability and selectivity for **4a** and **4b** changed only slightly compared to a PIM-1 membrane prepared similarly.

**Figure 4:**
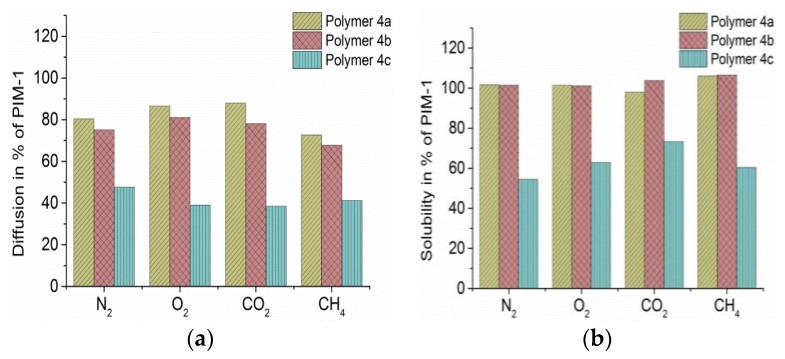
Diffusivity (**a**); and solubility (**b**) coefficients of polymers **4a–c**, calculated from time lag measurements at 30 °C and set in relation to PIM-1 membrane (see Table 3).

By differentiating time lag measurements in diffusivity and solubility, it was found that **4a** and **4b** had a slightly reduced diffusivity compared to PIM-1 but similar or even slightly enhanced (in case of CH_4_) solubility (Figure 4). These results explain quite well the observed permeability. Polymer **4c**, however, showed a serious reduction in permeability, and selectivity of CO_2_ over N_2_ was lower than with PIM-1 membrane. Despite its low content of PEO, it lost more than half of diffusivity and one third of the solubility compared to PIM-1 values (Figure 4). However, diffusivity of N_2_ is affected less than the other gases and this caused the lowered selectivity of CO_2_ and O_2_ on N_2_ compared to PIM-1. The long chain of PEO-5000 has an original crystallinity of more than 95%; the crystallinity of quenching compound **3c** is still at ca. 67%. From DSC measurements, a crystallinity of polymer **4c** of ca. 1% was calculated (after [21]).

To Summarize, the effect of the PEG-mono-esters (**3a–c**) as quenching compounds in polycondensation and on PIM-1 performance is quite limited. The low molecular PEG-compounds **3a** and **3b** react (quench) better because of their enhanced mobility in solution. Their achieved permeability and selectivity are quite comparable to PIM-1. PEG-5000-ester **3c** with its 100 (O–CH_2_–CH_2_) repetition units is too large to gain a similar degree of substitution to PEG-350 and PEG-750. It reduces permeability, diffusion and solubility considerably, but not in the desired direction.

### 3.3. Formation of PIM-1-b-PEO Multiblock Copolymers (Path 3)

**Scheme 2:**
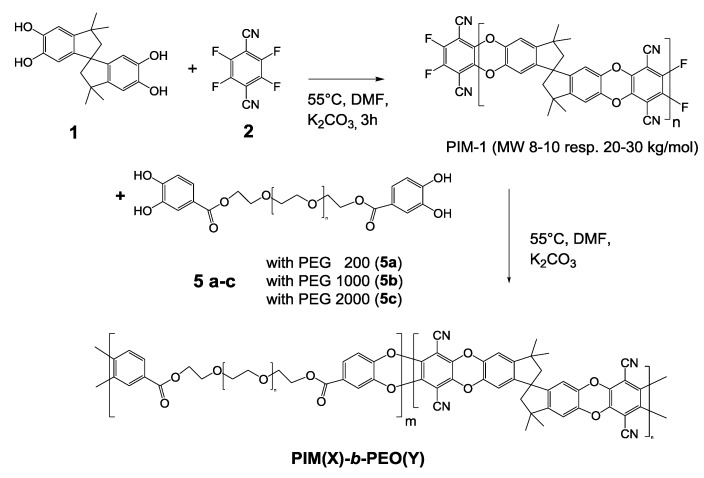
Preparation of **PIM(X)-*b*-PEO(Y)** multiblock copolymers in two reaction steps. Step 1: polycondensation with excess of **2** over **1** to achieve low molecular PIM-1 (1.1:1 = 8–10 kg/mol, and 1.025:1 = 20–30 kg/mol); and Step 2: polycondensation with DHBA-di-esters **5a–c**.

Within the polymers described in Path 2 (Section 3.2), the PIM-1 fraction is relatively large compared to the PEG part, e.g., 50 kg/mol versus 0.2–5 kg/mol. While the PIM-1 fraction is already a film forming polymer, the prepared PEO containing polymers represented a PIM-1 intercepted by more or less small inclusions of PEO. Unfortunately, PEGs of high molecular weight, e.g., 50 kg/mol, are not useful because of their high crystallinity and consequently very low permeability [10]. Therefore, on Path 3, we decided to reduce the chain length of PIM-1 to approximate the level of the used PEGs. Multiblock copolymers were formed by two consequent steps of polycondensation combining low molecular PIM-1 treated to end in fluoro groups and di-esters of dihydroxybenzoic acid formed with PEGs of different chain length (see Scheme 2).

**Figure 5:**
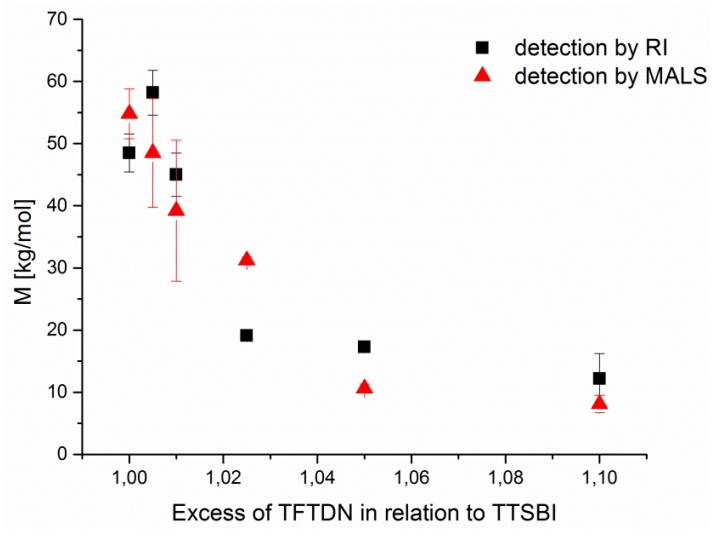
Molecular weight of PIM-1 prepared by polycondensation with excess of **2** above **1**. Reaction conditions: K_2_CO_3_ (2.04 molar towards **1**), 3 h at 55 °C in DMF. SEC measurements detected by RI (squares) and MALS (triangles).

The influence of excess of tetrafluoro-1,4-dicyanobenzene (**2**) on the molecular weight of the formed PIM-1 was examined in a series of polycondensations that ran exactly 3 h of reaction time at 55 °C under identical conditions. The resulting PIM-1 polymers were dissolved in CHCl_3_ and measured in SEC; achieved molecular weights were determined by two different detection modes: Refractive index (RI) and multi angle laser light scattering (MALS). The achieved molecular weights are depicted in Figure 5 as a function of the ratio of **2** versus **1** (detection: RI (squares) and MALS (triangles)). At an equimolar relation of hydroxy (OH) and fluoro (F)-groups and a moderate excess of K_2_CO_3_ (2.04 molar) the achieved molecular weight is in the range of 45 to 55 kg/mol with PDI of ca. 3, comparable to the results of Guiver et al.: *M*_w_ of 65 kg/mol and PDI of 2.2 at 60 °C within 3 h [22]. An increase of the relation F:OH up to 1.025:1 (2.5% excess of **2** above **1**) came out to about 20–30 kg/mol with PDI of 2.2 to 3.2. A relation of F:OH = 1.1:1 (10% excess of **2**) resulted in approximately 8–10 kg/mol, with PDI of 2. These results are fairly reproducible, by using the identical conditions, the respective molecular weight of the prepared PIM-1 can be directed by the ratio of F:OH monomers.

Subsequently, low molecular PIM-1 of defined molecular weight reacted in a successive second polycondensation step at 55 °C, with one of di-esters **5a–c** (Scheme 2) and catalyzed by an additional amount of K_2_CO_3_. Monomers **5a–c** were prepared from DHBA and poly(ethylene glycol) (200, 1000, and 2000 kg/mol) by standard method identical to monoesters in Section 3.2 (see Supplementary Materials S1. *Monomer synthesis example 2*). The slowly formed multiblock copolymers were named **PIM(X)-*b*-PEO(Y)**: X gives the approximately molecular weight of PIM-1, Y the molecular weight of the PEG as given by the manufacturer, both numbers representing kg/mol units. Progress of the reaction was followed by sampling and subsequent analysis in SEC (see below). The reactivity of the PEG- di-DHBA-esters **5a–c** as tetrahydroxy component in polycondensation reaction is definitely lower compared to **1** and therefore reaction times needed to obtain film forming polymers (*M*_w_ of at least 40 kg/mol) turned out to be longer than with PIM-1(1–14 days). Final work up of polycondensation reactions was by precipitation in water, filtering and drying at 60 °C (see Supplementary Materials S1. *Polymerization example 4*).

#### 3.3.1. SEC of PIM(25)-*b*-PEO(2)

**Figure 6:**
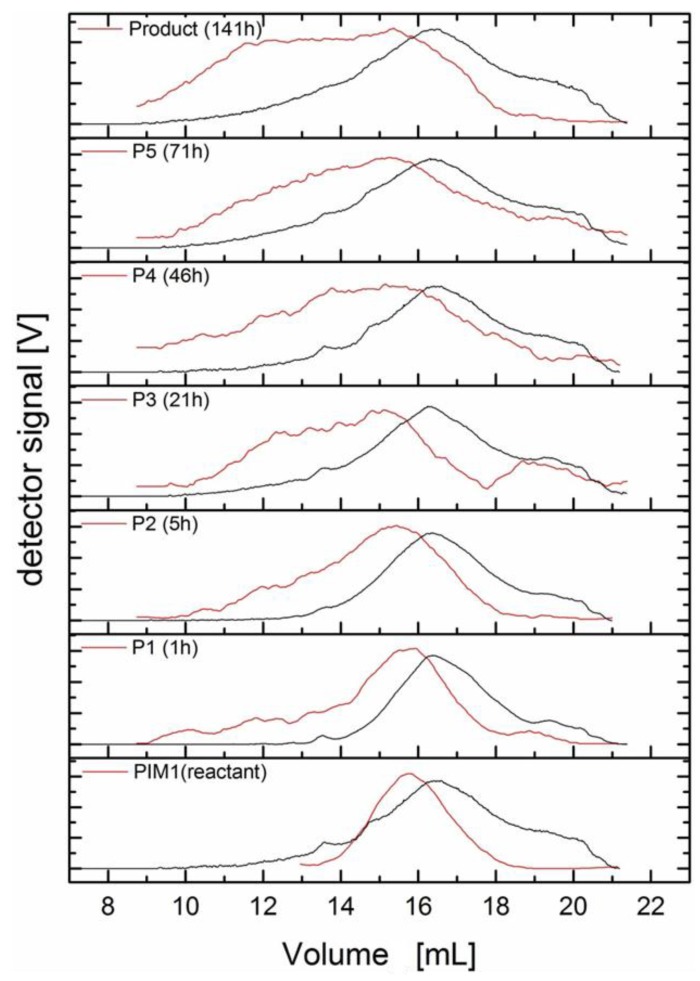
SEC during polycondensation of **PIM(10)-*b*-PEO(1)**; detection by RI (black lines) and by MALS (red lines).

In Figure 6 are depicted typical SEC measurements of samples taken during an extended poly-condensation resulting finally in **PIM(25)-*b*-PEO(2)** with rather high molecular weight. These samples were prepared by precipitation in water and worked up either by extraction with CHCl_3_ (samples from the start) or by direct filtration. All samples were measured in SEC (liquid phase CHCl_3_). Black lines show the eluted volumes, differentiated by reaction time and detected by RI, the red lines were results of MALS detection. The black line at the bottom of Figure 6 (PIM-1 reactant) represents the starting PIM-1 sample with F-end groups and approximately 20–25 kg/mol with a broad peak at 16–17 mL eluted volume. Monomer PEG-2000-di-DHBA-ester (**5c**) was added and after 60 min the next black line was measured. During progress of reaction the initial PIM-1 peak broadened towards lower elution volume (=higher molecular weights). Additional samples were drawn after 10 h, 45 h and 75 h of reaction time. Finally, the product is represented by the black line on top of Figure 6. Distribution of molecular weights is very broad. The final *M*_w_ measured by RI detection was calculated by universal calibration (polystyrene (PS)) standards to average 380 kg/mol with quite large deviations. However, a basic problem with RI detection is the universal calibration by polystyrene standards. The contorted structure of PIM-1 (and the similar polymers in our study), introduced by the spiro-formation of **1**, is the reason for the apparent intrinsic microporosity by hindering the polymer chains to pack efficiently and thus forming molecular nano-sized pores in between the chains. As the product of a polycondensation reaction, PIM-1 shows a rather broad mixture of different chain lengths (PDI >> 1), therefore many deviations towards higher and also lower molecular weights. Average molecular weight of polymers is usually estimated by SEC on calibrated separation columns and in most SEC labs the universal calibration by commercial available PS standards and detection by refraction index (RI) and/or UV signals is the acknowledged standard method. Separation on SEC columns also depends on the hydrodynamic volume of the polymers and PIM-1 with its contorted structure is quite different from PS. Therefore, this standard method provides not always reliable molecular weight values. However, from SEC measurements at different concentrations and detection by RI the determination of the refractive index (RI) increment dn/dc is available comparing the deviation from respective PS standards. Using the *d*_n_/*d*_c_ value measured for the employed polymers, it is possible to detect also by Multi Angle Light Scattering (MALS) (method see for example [23]) and to achieve more reliable molecular weight values for PIM-1 and the structural variations that are described in this study.

The red lines in Figure 6 display the detection of the identical samples by MALS, the final product (on top) was calculated to 130 kg/mol/PDI 1.1 by MALS using the measured value of *d*_n_/*d*_c_ = 0.174. The results for *M*_w_ and *M*_n_ obtained by MALS detection are in most cases more reasonable than those by RI detection. Although, during these experiments it was also observed that detection by MALS is of rather low sensitivity to molecular weights below ca. 15 kg/mol. The first two red lines in a comparable experiment to produce **PIM(10)-*b*-PEG(1)** (sample of low molecular PIM-1 and reaction after 60 min) show even lower intensities above 18 mL of elution volume compared to RI signals. The restricted sensitivity of the laser light for small polymer molecules explains also the rather low values of PDI (1.1 to 2.1) calculated from *M*_w_ and *M*_n_ estimated by MALS in comparison to PDI values of 3 to >10 calculated from RI detection. Therefore, it was decided to follow polycondensation reactions by a combination of RI and MALS to get the complete information.

All prepared PIM(X)-*b*-PEG(Y) multiblock copolymers were characterized by ^1^H-NMR, FT-IR, SEC with RI/MALS detector, TGA and DSC (see Supplementary Materials S1). In ^1^H-NMR the signal of PEO at 3.6 ppm is clearly visible and because of the relatively high PEO-diester content, compared to Path 2 in Section 3.2, also the aromatic protons of DHBA (>7 ppm) as well as the ester COOCH_2_ group at 3.8 ppm are detected. The integrals of the PIM-1 block (two singlets at 6.8 and 6.4 ppm representing the aromatic hydrogen of spirobisindane **1**) and of the PEO block (OCH_2_ at 3.6 ppm) are sufficiently separated to allow calculation of the approximate content of PEO within the polymers (see Table 4). All synthesized polymers are eventually film forming; SEC analysis (MALS) proved M_w_ to be in the range of 45 up to 70 kg/mol with PDI of 1.1–2.1 (Table 4).

The approximate PEO content of the multiblock copolymers was calculated also from TGA measurements: A 1st (minor) weight loss step up to 200 °C was caused by the loss of some adsorbed water and residual solvent, while the PEO-containing DHBA-diester block disintegrates in the 2nd step between 250 °C and 400 °C. Depending on the PEG chain included, the 2nd weight loss step was converted to the solely PEO content, e.g., the polymer block with monomer **5a** (PEG-200) contains 42% PEO, **5b** (PEG-1000) 78% and **5c** (PEG-2000) 88%. A 3rd step above 450 °C marked the final decomposition of the PIM-1block.

From DSC measurements the crystallinity was calculated (Table 4). Multiblock copolymers with PEO-200 showed a very low crystallinity below 0.1%, with PEO-1000 and PEO-2000 the crystallinity was still below 1%, which proves the quite even distribution of the blocks within the respective polymers.

All prepared multiblock-copolymers are sufficiently soluble in CHCl_3_ to allow the formation of dense membranes by solution casting. However, the resulting membranes are rather brittle and not as flexible as PIM-1. They required membrane thicknesses of 70 to 100 µm to withstand the pressure difference in time lag apparatus. Permeability and selectivity results of PIM-*b*-PEG multiblock copolymer membranes at 30 °C are presented in Table 5. A direct comparison to a respective PIM-1 membrane is not possible because neither PIM-1 of 10 nor 25 kg/mol is film forming. **PIM(10)-*b*-PEO(2)** was prepared solely to check the feasibility of the polycondensation reaction, the PEO content of 16–18 wt % is definitely too high for tests in gas separation and these membranes are therefore not included in Table 5.

**Figure 7:**
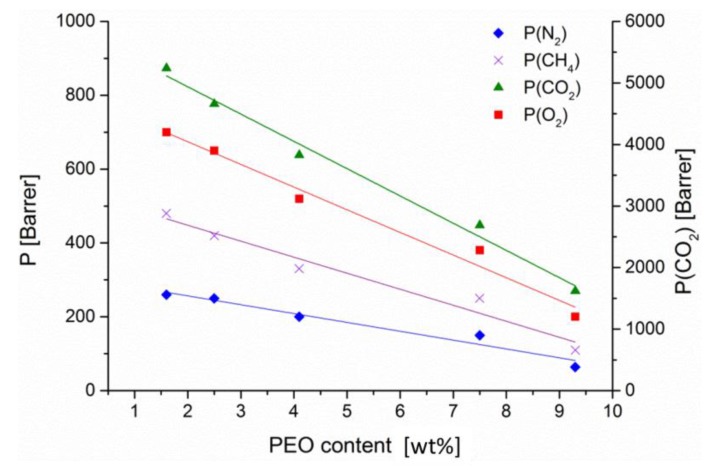
Permeability of 4 different gases depicted versus PEO content (values from TGA, see Table 4) in PIM-*b*-PEO multiblock copolymers, measured in time lag apparatus at 30 °C. Left: Permeability of N_2_, O_2_, CH_4_; right: CO_2_.

In Figure 7, permeability of the usual set of four gases is depicted versus the approximate PEO content and turned out to be in good linear dependency. A graphical extension to 0% of PEO leads to quite reasonable permeability of 300 (N_2_), 800 (O_2_), 5800 (CO_2_) and 500 (CH_4_) Barrer for the hypothetical PIM-1 block and matches approximately the values of PIM-1 membrane mentioned in Table 2.

**PIM(25)-*b*-PEO(0.2)** represents the lowest PEO content (ca. 1 wt %) and showed rather high permeabilities, e.g., more than 5000 Barrer of CO_2_. The separation behavior is also similar to PIM-1 with separation factors of 20 for CO_2_ over N_2_ and 10.9 for CO_2_ over CH_4_. With rising content of PEO up to 7–9 wt % permeability decreased to about a half of the 1 wt % PEO value but selectivity at higher PEO-contents increases only slightly (see Table 5, right side). Differentiating the time lag measurements into diffusion and solubility gives the already expected result that diffusion is linearly reduced with rising PEO content for all four measured gases. Solubility coefficients, however, stayed almost the same with O_2_ and N_2_ (ca. 0.02), while the solubility of CH_4_ (0.1–0.12) as well as solubility of CO_2_ (0.50–0.60 cm^3^/(cm^3^ bar)) increased slightly.

### 3.4. Path 4: PEG as Side Chain in PIM-1-Copolymer

To introduce a PEG side chain into a PIM-1-copolymer we used the monomer tetrahydroxy-9,10-dibutylanthracene (**6**) as vehicle and added maleimides to **6** in a similar chemistry as published recently by Khan et al. [14]. **6** is easy to prepare, although in low yield (ca. 25%), from 1,2-dimethoxybenzene and pentanal following the synthesis described in [23] using pentanal instead of acetaldehyde. It shows good solubility in many common solvents, caused presumably by the butyl substitution in 9,10-position. Methyl substitution in the related tetrahydroxy-9,10-dimethylanthracene resulted in a hardly soluble component [14].

**Scheme 3:**
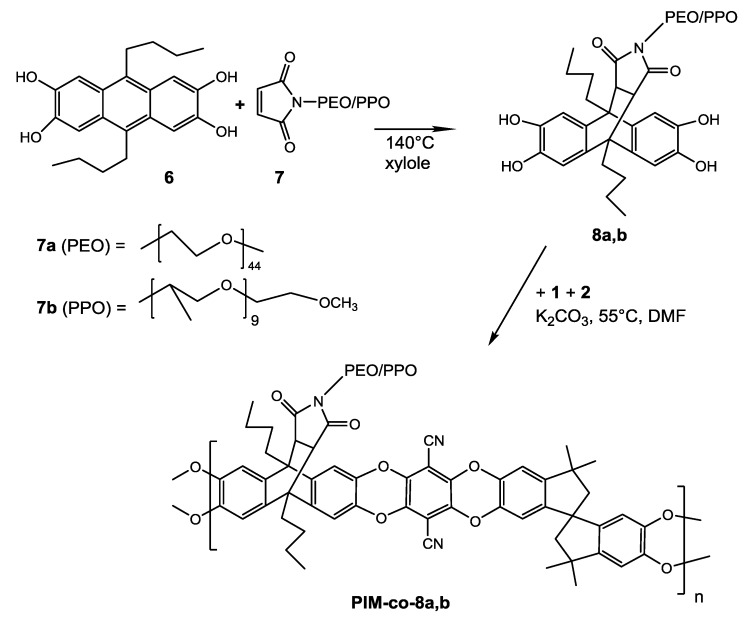
Preparation of tetrahydroxy-monomers **8a** and **8b**, and in the following polycondensation to **PIM1-co-8a** and **PIM1-co-8b** (55 °C in DMF/3–5 days).

By Diels–Alder-reaction at elevated temperature anthracene **6** offers the possibility to add a dienophilic substituted double bond (see Scheme 3). Maleimide-(PEO)_44_ **7a** ( Specific Polymers, Castries, France) is commercially available and has a molecular weight of 2032 g/mol providing a PEO chain of 1936 g/mol. The maleic double bond adds successfully to tetrahydroxy-monomer **6** at 140 °C in 24 h and with a good yield (90%). The product of cycloaddition, monomer **8a**, was characterized by ^1^H-NMR, IR, TGA and DSC (see Supplementary Materials S1); it shows a distinctive melting point of 46 °C determined by the long PEO side chain.

Tetrahydroxy-monomer **8b** was prepared analogously by Diels–Alder addition of maleimide-methoxypolypropylene glycol (**7b**), with a PPO chain of ca. 600 g/mol, to **6** (see Scheme 3). **7b** was synthesized by a low temperature method [24] from commercially available Jeffamine^®^-M600 (Huntsman, Germany) and maleic anhydride. It has a molecular weight of 677 g/mol and adds to **6** at similar conditions as mentioned above. **8b** was obtained in 70% yield as highly viscous oil and was also fully characterized by ^1^H-NMR, FT- IR, TGA and DSC (see Supplementary Materials S1).

Both components **8a** and **8b** were successfully employed as comonomers in PIM-1 polycondensation in various relations to **1**. Reaction conditions are similar to PIM-1 polycondensation but with prolonged reaction times of at least three days at 55 °C to achieve a film forming polymer (see Supplementary Materials S1. *Polymerization example 5*). Introduction of the long hydrophilic PEO chain changes the quality of the (co)polymer decisively, e.g., copolymers with content of **8a** higher than 40 wt % became in fact water soluble and too soft for membrane formation. However, the experience of the Paths 1–3 suggested a desirable PEG content below 10 wt % to avoid exceeding reduction of gas permeability. Therefore it required only 2.5 mol-% of **8a** for tetrahydroxy component together with **1** in polycondensation with **2** to obtain a calculated overall PEO content of 9.4 wt %. The melting point of the long PEO chain of **8a** was also observed in DSC measurement of the formed copolymer **PIM1-co-8a-1**.

Within copolymer **PIM1-co-8a-1** averagely every 40th molecule of **1** is substituted by **8a**. Nevertheless, in FT-IR the O-C stretching at 1100 cm^−1^ can be seen perfectly, filling in a gap within the PIM-1 spectrum. ^1^H-NMR spectrum shows the prominent OCH_2_ singlet at 3.64 ppm, but because of the low molar quantity of **8a** only OCH_2_ is visible, while the aromatic and aliphatic hydrogen peaks of **8a** are overlaid by PIM-1. For reasons of comparison and without any impact of **8a** the singlet of PIM-1 at 6.42 ppm was suited best for calculation of composition, integration estimates the PEO content to 2.7 mol %. From the 1st step of decomposition in TGA at an onset of 377 °C, about 9.1 wt% weight loss was identified that represents the Retro-Diels–Alder reaction splitting off the maleimide-PEO **7a**, corrected to PEO a content of 9 wt % was estimated. SEC with MALS detection (*d*_n_/*d*_c_ = 0.2611) revealed molecular weight of ca. 40 kg/mol and PDI of 1.4. Transparent membranes were formed from CHCl_3_ solutions of this copolymer; they are quite stable and were measured in time lag apparatus with the usual set of four gases (results in Table 6).

Monomer **8b** contains a shorter chain of about 600 g/mol that additionally introduce a branching originating from its polypropylene glycol (PPO) basis. It should be less hydrophilic than poly(ethylene glycol). It was added in 5 and 10 mol % of tetrahydroxy component in PIM-polycondensation, resulting in copolymers of 6 and 11 wt% calculated PPO content (**PIM1-co-8b-1** and **PIM1-co-8b-2**), respectively. In ^1^H-NMR spectrum the multiplet between 3.36 and 3.6 ppm covers OCH_2_, CH as well as OCH_3_ signals within the PPO-chain. Compared to the singlet of the PIM-1unit at 6.42 ppm, the PPO content was calculated to 4.5 mol % (**PIM1-co-8b-1**) and 8.8 mol % (**PIM1-co-8b-2**). Furthermore, TGA measurements include the Retro-Diels–Alder reaction as the loss of maleimide **7b** (observed as a first weight loss step below 400 °C), re-calculation to PPO content yielded 6.0 wt % PPO in **PIM1-co-8b-1** and 8.6 wt % PPO in **PIM1-co-8b-2**, respectively. These latter values are included in Table 6. Both new copolymers dissolve easily in CHCl_3_. SEC was measured with MALS detection to 46 kg/mol and 42 kg/mol, with PDI = 1.2 for both polymers. By casting from CHCl_3_ solution, stable membranes could be formed.

**Figure 8:**
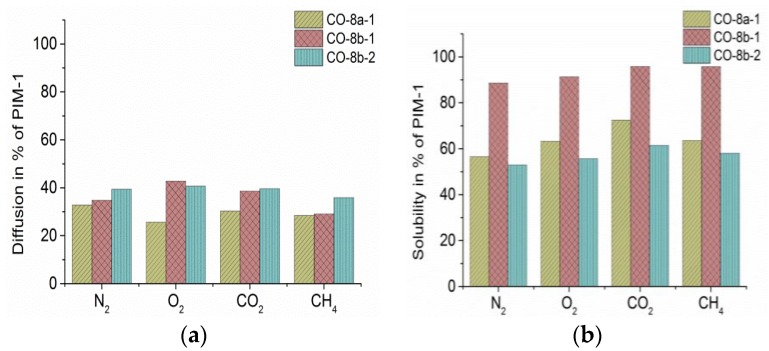
Diffusivity (**a**); and solubility coefficients (**b**) of polymers **PIM-CO-8**, calculated from time lag measurements at 30 °C and in relation to pristine PIM-1 membrane (in Table 2).

Gas permeability of PIM-copolymers with PEO and PPO in side chain was measured at 30 °C in time lag apparatus; results are presented in Table 6. Caused by the quite high PEO/PPO content of 6–9 wt %, permeability is low and selectivity is enhanced compared to a pristine PIM-1 membrane, as presented in Table 2. The difference between PEO and PPO is not very pronounced when considering that a PPO chain of 600 g/mol in **8b** is rather short compared to the PEO chain of ca. 2000 g/mol in **8a**. The low permeability is caused primarily by a reduction to 30–40% diffusivity (see Figure 8a, directly compared to PIM-1 value given in Table 2), as was already observed within Path 1. Solubility is affected less, especially for CO_2_ and CH_4_ (Figure 8b).

## 4. Summary of All Approaches

Permeability of PIM-1 membrane in gas separation depends on several variables such as process of preparation and casting solvent [22], as well as pre-/treatment [25]. Values, e.g., for CO_2_ permeability at 20–30 °C have been presented in the literature, ranging from 2300 Barrer [4] to 12,600 Barrer [5]. We also observed the phenomenon that was already mentioned by Guiver et al. [22] that PIM-1 membranes prepared from CHCl_3_ or CH_2_Cl_2_ have definitely higher permeability than those made from tetrahydrofurane or dichlorobenzene under otherwise identical conditions. To exclude differences on this account, all membranes in our study were cast from the same solvent and, fortunately, we could use CHCl_3_ for all membranes. As with most high flux glassy polymers, a severe ageing/compaction over time takes place reducing permeability significantly within a rather short measure of time [26]. Actually, we measured permeability of N_2_ and O_2_ of several membrane examples from our study for at least 100 h but observed a quite similar ageing behavior like PIM-1. However, ageing of PIM was not the task of our study and would have gone beyond the scope of this article.

Apparently, it is all together no easy task to define a base line to rank newly developed membranes on PIM-1 basis. In this study, we referred to PIM-1 mostly to directly measure equivalent values (Path 1) or chose a fresh batch of PIM-1 prepared under identical conditions (Paths 2 and 4). In the case of Path 3, we had no reference value; therefore, we referred to the lowest concentration of PEO and to the graphical reduction of measured permeability to 0% of PEO. All membranes were measured as soon as possible after casting to avoid a noticeable influence of ageing effects.

The outstanding of the four gases examined in this study with new combinations of PIM-1 and PEG and PEO/PPO is CO_2_, displaying the highest permeability because of a high solubility in PIM-1 as well as in PEG. The other measured gases, N_2_, O_2_, and CH_4_, follow the behavior of CO_2_ on regard of diffusivity on a similar level, but they are much less soluble.

**Figure 9:**
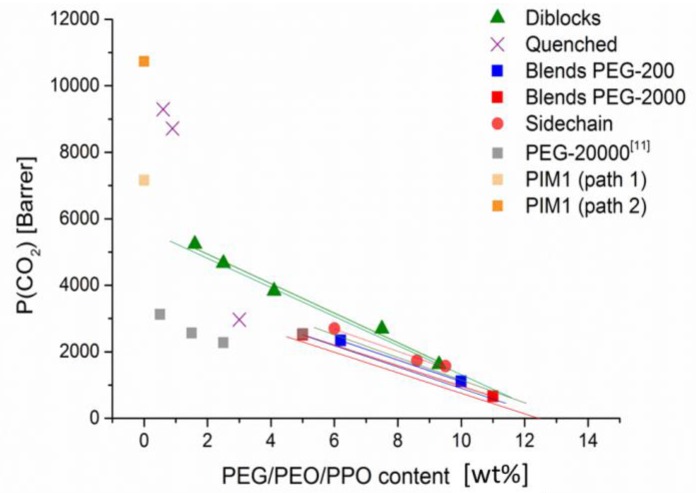
Graphical summary of all approaches compares measured CO_2_ permeability (time lag, single gases, 30 °C) versus PEG and PEO/PPO-content from this work: blends with PEG-200 and PEG-2000 (dark squares), quenched polymers (crosses), multiblock-copolymers (triangles), and side chain-copolymers (circles). Data from [11] are also included: Blends of PIM-1 with PEG 20 kg/mol (pale squares).

In Figure 9, a summary of all paths is presented, comparing CO_2_ permeability of PEG/PEO/PPO containing membranes by means of the actual PEG/PEO/PPO content. Addition of trend lines leads the eyes and allows consequently comparison of all approaches to combine PIM-1 and PEG/PEO/PPO. Two data points for pristine PIM-1 permeability are included (from Table 2 and Table 3), these both already displaying the variety of measured CO_2_ permeability of PIM-1 membranes under research. In Figure 9, it is obvious that every substantial addition of PEG or PEO to PIM-1 leads to a linear reduction in membrane permeability, caused by the low permeability of PEG(s) in comparison to the superior properties of PIM-1. Differences within the paths are diminishing with increase of PEO content towards and above 10 wt %. An amount of PEO above 10 wt % reduces permeability of CO_2_ clearly below 1000 Barrer and consequently the other gases in our study were reduced below 100 Barrer. Nevertheless, the effect of an increased hydrophilicity of produced membranes is noticeable in all four paths.

The very low addition (<<1 wt %) in case of Path 2 (Figure 9, crosses) seems not substantial enough to change the permeability of PIM-1 noticeably. Without the quenching by dihydroxybenzoic esters **3a–b** but simply precipitating in H_2_O membranes made of this PIM-1 batch showed about 10,000 Barrer of CO_2_ and a selectivity of ca. 19 for CO_2_/N_2_ and that did not change by quenching with **3a–b** (Table 3). The high values of permeability of these membranes are achieved by a short reaction time in polycondensation and a rather low molecular PIM-1 of ca. 50 kg/mol. By adding a large PEO chain like PEG 5000 (**3c**) resulting in polymer **4c** the influence of PEO on permeability becomes definitely visible by a severe decrease to less than 3000 Barrer CO_2_. The low selectivity of polymer **4c** for CO_2_/N_2_ of ca.16, however, points to a similar phase separation by extended regions of PEG as in [11] when a blend of PIM-1 with 2.5 wt % PEG-20000 (pale squares) has a still quite high permeability of CO_2_ of ca. 2300 Barrer but also a rather high permeability of N_2_ of 140 Barrer. Although, the membranes prepared from polymer **4c** were optically rather transparent.

Preparation of blends of PIM-1 and different PEGs (Path 1, Figure 9, dark squares) is surely the simplest method to combine PIM-1 and PEG. Membranes prepared from the physical mixtures within Path 1 behave quite similar: With increasing PEG content, permeability of all measured gases decreased and selectivity of CO_2_/N_2_ increased slightly (from 18 up to 30). Influence of PEG chain length of the employed PEGs between 200 and 2000 g/mol in our study is not very pronounced and can be distinguished merely in high resolution of the graphs. Wu et al. [11] presented only one blend membrane with a content as high as 5 wt % but prepared with PEG-20000 (Figure 9, pale squares). Permeability of CO_2_ of this membrane (2550 Barrer) is comparable to our own blend with 5 wt % PEG-2000 (2500 Barrer, Figure 9, dark square) but permeability of N_2_ (200 Barrer) is higher with PEG-20,000, resulting in a selectivity of CO_2_/N_2_ = 12, compared to 25 with PEG-2000 (N_2_ permeability 100 Barrer) in our study. Their experiments in consequence concentrated on low amounts of PEG-20000 to save a better part of PIM-1 permeability. It should be mentioned that they used a PIM-1 of lower initial permeability, which explains the shift to lower permeability in Figure 9 (pale squares) compared to our results. Nevertheless, they achieved lower selectivity of CO_2_ with respect to N_2_ in the range of 16–12, obviously reducing N_2_ permeability less than CO_2_ by addition of high molecular PEG. This could be caused by the formation of separated phases within the membranes forming nanochannels that discriminate only larger molecules like CH_4_. The remarkable found of their work indeed was an increase of selectivity for CO_2_ over CH_4_ up to 39, compared to a selectivity of 10–12 of pristine PIM-1. The low molecular PEGs in our work allow a continuous membrane phase in most cases with a similar reduction of all measured gases in diffusion, compensated by an only moderate reduction of solubility of the highly soluble CO_2_. Therefore, selectivity of CO_2_/CH_4_ is influenced only moderately up to a value of 14, except the blend with PEG-2000 that reached a value of 18.

Graphical prolongation of the trend lines of our experiments with PEG-200 and PEG-2000 to 0% of PEG leads to 4000 to 4300 Barrer of CO_2_, while the original PIM-1 batch had shown a permeability of more than 7000 Barrer. The dependency of P on addition of PEG and PEO seems to be non-linear at low amounts as the experience of Path 2 already pointed to.

Multiblock copolymers **PIM(x)-*b*-PEO(Y)** contain PIM-1 parts of quite low molecular weight (10 and 25 kg/mol) and blocks of even smaller PEO (0.2, and 1.2 kg/mol), but these blocks are divided evenly among the polymer chain without a chance of phase formation. Pure PIM-1 polymers of low molecular weight like that are not film forming therefore no comparable membranes could be provided. In Figure 9 multiblock copolymers (triangles) show the highest permeability of all paths on regard of the PEO content. At ca. 5 wt % of PEO a multiblock copolymer membrane would show 3500 Barrer of CO_2_, about 1000 Barrer more than a membrane made from PIM-1-PEG blend of similar PEG chain length. A graphical prolongation to 0% PEO achieved about 5600 Barrer CO_2_ of the remaining PIM-1 polymer, a value definitely higher than that calculated from the PEG blended membranes in Path 1.

PEO and PPO introduced as a side chain was described in Path 4, using the different co-monomers **8a** and **8b** to partly substitute tetrahydroxyspirobisindane (**1**) in PIM-1 polycondensation. The roof shaped bridged anthracene introduced an additional bend angle to the already contorted PIM-1 chain as described in [27]. PEO and PPO side chains fixed to the ethane bridge adding up to 6 to 10 wt% PEO and PPO within the copolymers **PIM1-co-8a** and **PIM1-co-8b**, respectively. Obtained permeability is located within the values measured on Path 1 and Path 3. PEO and PPO chains are of limited mobility as in Path 3 but pure PIM-1 parts within are quite large and therefore these polymers are more comparable to Path 1.

**Figure 10:**
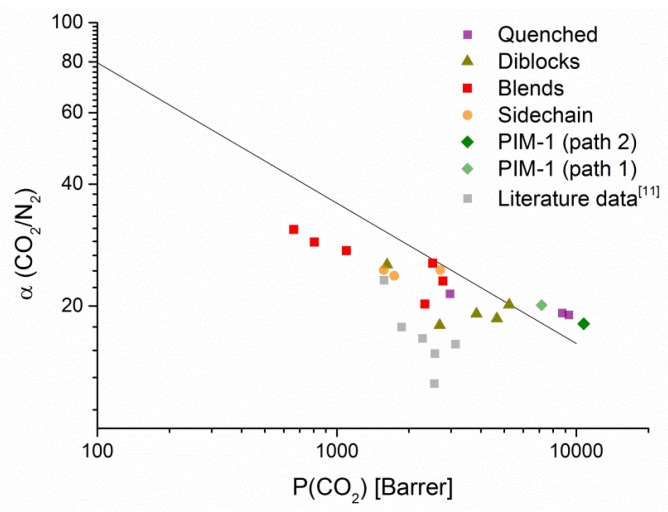
Selectivity of CO_2_/N_2_ versus permeability of CO_2_ at 30 °C: blends with PEG-200/-2000 (dark squares), quenched polymers (crosses), multiblock-copolymers (triangles), side chain-copolymers (circles), and, for comparison data, from [11]: Blends of PIM-1 with PEG 20 kg/mol (pale squares). The depicted section of Robeson’s upper bound for CO_2_/N_2_ was extracted from [6].

In Figure 10 is depicted exemplarily a section from Robeson’s plot (the “upper bound” of 2008 [6] represented by the black line) for selectivity of CO_2_/N_2_ versus permeability of CO_2_ at 30 °C. Included are only PIM-1 values from our study, one of them is above the upper bound, one below. Permeability of our PIM-1–PEO combinations is reduced compared to the pristine PIM-1 and therefore, following Robeson’s trade-off, an increase in selectivity for CO_2_/N_2_ distinctively above 20 is achieved in most cases, however, by relinquishing a considerable part of permeability (Figure 9). Diagrams made up for CO_2_/CH_4_ (increase in selectivity from 10 up to 14–18), or O_2_/N_2_ (increase in selectivity from 2.8 up to 3.5) (neither shown) provide identical information but on a much lower (as well permeability as selectivity) level.

## 5. Conclusions

In our study, we tested four different paths to combine PIM-1 with up to 10 wt % PEG and PEO/PPO to enhance the hydrophilicity of the mixed polymer considerably. Physical mixtures and new, chemically linked polymers were successfully formed into free standing dense membranes and measured in single gas separation of N_2_, O_2_, CO_2_ and CH_4_ in time lag method. Melting of long PEG chains (2–5 kg/mol) at elevated temperatures up to 80 °C had no observable influence on permeability; further measurements were performed at 30 °C.

Permeability was lowered by any substantial addition of PEG/PEO/PPO, almost regardless the manufacturing process and quite proportional to the added amount. It can be stated that roughly about 6 to 7 wt % of PEG/PEO/PPO in PIM-1 combination halved the permeability of the original PIM-1 membrane. Selectivity calculated from single gas measurements increased slightly up to values of about 30 for CO_2_/N_2_, a maximum of 18 for CO_2_/CH_4_ and 3.5 for O_2_/N_2_. In all combinations, PIM-1 is the highly permeable part and obtained gas fluxes are defined by the amount and the distribution of PEG and PEO. Blending of PIM-1 and PEGs of different chain length led to phase separation at higher concentrations of PEGs and an even lower selectivity than with PIM-1. An even distribution within the multiblock copolymers **PIM(X)-*b*-PEO(Y)** resulted in slightly higher permeability at similar concentration than a PEO part with a long chain, as in **4c** or **PIM1-co-8a**.

By differentiating permeability in diffusivity and solubility, it was shown that addition of PEG/PEO/PPO affected diffusivity of all measured gases quite uniformly. Solubility of CO_2_, O_2_, and CH_4_, however, was reduced less than solubility of N_2_ resulting in increased selectivity for the former gases. Concentration optimum for the combination of PIM-1 and PEO between gaining solubility for selected gases and losing not too much permeability for all gases under consideration is in the range of 2–5 wt % PEO.

For perspective, these modified PIM-1 membranes of enhanced hydrophilicity should be useful in other membrane techniques such as nanofiltration and pervaporation, by combining the very good chemical resistance of PIM-1 with a selective permeance because of a higher hydrophilicity. Addition and even distribution of nanoparticles or other more polar fillers should also be facilitated in presence of PEG chains.

## Figures and Tables

**Table 1:** Description and schematic (not true to scale) presentation of the four different approaches to combine PEG and PEO (ellipsoids) and PIM-1 (rectangles), physically mixed or chemically bound. Anthracene based copolymer (Section 3.4, Path 4) is depicted as triangle to distinguish from PIM-1.

Path 1 (Section 3.1)	Path 2 (Section 3.2)	Path 3 (Section 3.3)	Path 4 (Section 3.4)
Blend membranes from PIM-1 and different low molecular PEGs	Quenching to form PEO end groups of PIM-1 chains	PIM1(X)-*b*-PEO(Y)—multiblock copolymers	PEO/PPO in side chain of PIM1 copolymer
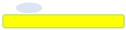	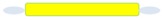	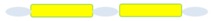	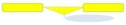

**Table 2:** Time lag measurements at 30 °C of four gases using membranes formed by blending PIM-1 with different PEGs.

	PEG-Content wt % ^d)^	Permeability (Barrer ^b)^)				Selectivity ^c)^		
		N_2_	O_2_	CO_2_	CH_4_	O_2_/N_2_	CO_2_/N_2_	CO_2_/CH_4_
PIM-1 ^a)^	0	360	1030	7160	590	2.8	19.9	12.1
+ PEG-200	6.2	115	310	2335	199	2.7	20.2	11.7
+ PEG-1000	5.3	120	361	2776	211	3.0	23.0	13.1
+ PEG-2000	5.0	100	314	2513	177	3.2	25.5	14.2
+ PEG-200	10.0	40	122	1097	78	3.0	26.8	14.1
+ PEG-1000	12.2	28	95	806	46	3.3	28.4	17.6
+ PEG-2000	11.0	20	74	660	37	3.5	30.9	18.0

^a)^ *M*_w_ ca. 300 kg/mol (SEC/MALS detector); ^b)^ 1 × 10^−10^ (cm^3^(STP)cm cm^−2^ s^−1^cm Hg^−1^); ^c)^ Calculated from single gas measurements; ^d)^ Content of PEG as calculated from TGA.

**Table 3:** Time lag results at 30 °C for PIM-PEG-polymers **4a–c** synthesized by quenching with PEG-esters of trihydroxybenzoic acid (**3a–c**) and compared to a freshly prepared PIM-1 membrane synthesized along the first part of Scheme 1.

	PEG-Content wt % ^a,b)^	N_2_	O_2_	CO_2_	CH_4_	O_2_/N_2_	CO_2_/N_2_	CO_2_/CH_4_
PIM-1 (50 kg/mol)	0	530	1480	10,000	900	2.8	18.8	10.9
Polymer (**4a**)	0.6 (0.5)	490	1360	9270	830	2.8	18.9	11.2
Polymer (**4b**)	0.9 (0.9)	450	1265	8700	780	2.8	19.2	11.2
Polymer (**4c**)	3.1 (2.6)	140	370	2210	270	2.7	15.8	8.2

^a)^ Because of TGA measurements of the 1st decomposition step; ^b)^ In brackets: Calculated by ^1^H-NMR integrals.

**Table 4:** PEO content by ^1^H-NMR and TGA, molecular weight of film forming polymers by SEC and MALS detection, and measured crystallinity of multiblock copolymers PIM(X)-*b*-PEG(Y) from DSC.

Multiblock Copolymer ^a,b)^	Intended PEO Content wt %	PEO Content (^1^H-NMR) wt %	PEO Content (TGA) wt %	*M*_w_ (MALS) kg/mol	PDI	Calc. Crystallinity (DSC) %
**PIM(10)-*b*-PEO(0.2)**	2	2.5	2.5	n.m.		0.06
**PIM(10)-*b*-PEO(1)**	9	10.8	9.3	67	1.3	0.7
**PIM(10)-*b*-PEO(2)**	16	n.m.	18	41	2.1	n.m.
**PIM(25)-*b*-PEO(0.2)**	1	1.2	1.6	57	1.1	0.08
**PIM(25)-*b*-PEO(1)**	4	3.6	4.1	43	1.3	0.11
**PIM(25)-*b*-PEO(2)**	7	7	7.5	44	1.1	0.14

^a)^ *M*_w_ of PIM-1 was roughly estimated by reaction time and excess of TFTDN (see Figure 5); ^b)^ *M*_w_ of PEG as given by deliverer.

**Table 5:** Permeability and selected selectivity of PIM-*b*-PEG multiblock copolymers of different PEO content from time lag measurements with four gases at 30 °C.

Multiblock Copolymers	PEG wt %	Permeability (Barrer)				Selectivity		
		N_2_	O_2_	CO_2_	CH_4_	CO_2_/N_2_	O_2_/N_2_	CO_2_/CH_4_
**PIM(25)-*b*-PEO(0.2)**	1.6	260	700	5240	480	20.1	2.7	10.9
**PIM(10)-*b*-PEO(0.2)**	2.5	250	650	4660	420	18.6	2.6	11.1
**PIM(25)-*b*-PEO(1)**	4.1	200	520	3830	330	19.1	2.6	11.6
**PIM(25)-*b*-PEO(2)**	7.5	150	380	2690	250	18.0	2.5	10.8
**PIM(10)-*b*-PEO(1)**	9.3	140	370	3000	270	22.3	2.8	11.1

**Table 6:** Time lag measurements of PIM-copolymers **8a,b** with PEO and PPO in side chain at 30 °C.

Polymer	PEO/PPO-Content (TGA) wt%	Permeability (Barrer)				Selectivity		
		N_2_	O_2_	CO_2_	CH_4_	O_2_/N_2_	CO_2_/N_2_	CO_2_/CH_4_
**PIM-co-8b-1**	6.0	110	400	2650	160	3.6	24.1	16.5
**PIM-co-8b-2**	8.6	73	230	1735	123	3,2	23.8	14.1
**PIM-co-8a-1**	9.0	64	190	1570	107	2.9	24.5	14.7

## Supplementary Materials

The following are available online at http://www.mdpi.com/2077-0375/7/2/28/s1, S1: Synthesis of Monomers and Polymers, S2: Gas Separation.

## References

[1] Carta M., Clarizia G., Jansen J.C., McKeown N.B. (2014). Gas Permeability of Hexaphenylbenzene Based Polymers of Intrinsic Microporosity. Macromolecules.

[2] Budd P.M., Makhseed S., McKeown N.B., Msayib K.J., Tattershall C.E. (2004). Polymers of intrinsic microporosity (PIMs): Robust, solution-processable, organic nanoporous materials. Chem. Commun..

[3] Budd P.M., Elabas E.S., Ghanem B.S., Makhseed S., McKeown N.B., Msayib K.J., Tattershall C.E., Wang D. (2004). Solution processed, organophilic membrane derived from a polymer of intrinsic microporosity. Adv. Mater..

[4] Budd P.M., Tattershall C.E., Ghanem B.S., Reynolds K.J., McKeown N.B., Fritsch D. (2005). Gas separation membranes from polymers of intrinsic microporosity. J. Membr. Sci..

[5] Budd P.M., Ghanem B.S., Msayib K.J., Fritsch D., Starannikova L., Belov N., Sanfirova O., Yampolskii Y., Shantarovich V. (2008). Gas permeation parameters and other physicochemical properties of a polymer of intrinsic microporosity: Polybenzodioxane PIM-1. J. Membr. Sci..

[6] Robeson L.M. (2008). The upper bound revisited. J. Membr. Sci..

[7] Brinkmann T., Pohlmann J., Wind J., Wolff T., Esche E., Müller D., Wozny G., Hoting B. (2015). Pilot scale investigations of the removal of carbondioxide from hydrocarbon gas streams using poly(ethyleneoxide)–poly(butylene terephthalate)PolyActiveTM) thin film composite membranes. J. Membr. Sci..

[8] Freeman B. (2011). Membrane Gas Separation.

[9] Car A., Yave W., Peinemann K.-V. (2008). PEG modified poly(amide-b-ethylene oxide) membranes for CO_2_ separation. J. Membr. Sci..

[10] Freeman B.D., Lin H. (2004). Gas solubility, diffusivity and permeability in poly(ethylene oxide). J. Membr. Sci..

[11] Wu X.M., Peng J.L., Qu Y., Zhu A.M., Liu Q.L. (2015). Towards enhanced CO_2_ selectivity of the PIM-1 membrane by blending with polyethyleneglycol. J. Membr. Sci..

[12] Metz J., Wessling M. (2004). Gas-Permeation Properties of Poly(ethylene oxide) Poly(butyleneterephthalate) Block Copolymers. Macromolecules.

[13] Lillepärg J., Shishatskiy S. (2014). Stability of blended polymeric materials for CO_2_ separation. J. Membr. Sci..

[14] Khan M.M., Neumann S., Rahman M.M., Abetz V., Filiz V. (2014). Synthesis, characterization and gas permeation properties of anthracene maleimide-based polymers of intrinsic microporosity. RSC Adv..

[15] Wijmans J.G., Baker R.W. (1995). The solution-diffusion model: A review. J. Membr. Sci..

[16] Al-Ismaily M., Kruczek B. (2012). A shortcut method for faster determination of permeability coefficient from time lag experiments. J. Membr. Sci..

[17] Mason C.R., Al-Harbi N.M., Budd P.M., Bernardo P., Bazzarelli F., Clarizia G., Jansen J.C. (2011). Polymer of Intrinsic Microporosity Incorporating Thioamide Functionality: Preparation and Gas Transport Properties. Macromolecules.

[18] Fritsch D., Carta M., McKeown N.B. (2011). Synthesis and Gas Permeation Properties of Spirobischromane-Based Polymers of Intrinsic Microporosity. Macromol. Chem. Phys..

[19] Vopička O., Du N., Li N., Guiver M.D., Sarti G.C. (2014). Mixed gas sorption in glassy polymeric membranes: II. CO_2_/CH_4_ mixtures in a polymer of intrinsic microporosity (PIM-1). J. Membr. Sci..

[20] Li Y., Huang C., Liu G. (2013). Crystallization of poly(ethylene glycol) in poly(methyl methacrylate) networks. Mater. Sci..

[21] Fan W., Tian W., Zhu X., Zhang W. (2014). Differential analysis on precise determination of molecular weight of triblock copolymer using SEC/MALS and MALDI-TOF MS. Polym. Test..

[22] Song J., Dai Y., Robertson G.P., Guiver M.D., Thomas S., Pinnau I. (2008). Linear High Molecular Weight Ladder Polymers by Optimized Polycondensation of Tetrahydroxytetramethylspirobisindane and 1,4-Dicyanotetrafluorobenzene. Macromolecules.

[23] Balaban T.S., Krische M.J., Lehn J.-M. (2006). Hierarchic Supramolecular Interactions within Assemblies in Solution and in the Crystal of 2,3,6,7-Tetrasubstituted 5,5′-(Anthracene-9,10-diyl)bis[pyrimidin-2-amines]. Helv. Chim. Acta.

[24] Mehta N.B., Lui F.F., Brooks R.E. (1960). Maleamic and Citraconamic Acids, Methyl Esters, and Imides. J. Org. Chem..

[25] Jue M.L., McCool B.A., Finn M.G., Lively R.P. (2015). Effect of Nonsolvent Treatments on the Microstructure of PIM-1. Macromolecules.

[26] Swaidan R., Litwiller E., Pinnau I. (2015). Physical Aging, Plasticization and Their Effects on Gas Permeation in “Rigid” Polymers of Intrinsic Microporosity. Macromolecules.

[27] Emmler T., Fritsch D., Budd P.M., Chaukura N., Ehlers D., Raetzke K., Faupel F. (2010). Free Volume Investigation of Polymers of Intrinsic Microporosity (PIMs): PIM-1 and PIM1 Copolymers Incorporating Ethanoanthracene Units. Macromolecules.

