# Supplementary material for: Membranes of Polymers of Intrinsic Microporosity (PIM-1) Modified by Poly(ethylene glycol)
Source: Membranes (Basel). 2017 Jun 5;7(2):28. doi: 10.3390/membranes7020028 (PMC5489862; doi:10.3390/membranes7020028)
Supplement: Supplementary file 1 [file membranes-07-00028-s001.pdf]

Supplementary Materials: Membranes of Polymers of Intrinsic Microporosity (PIM-1) Modified by Poly(ethylene glycol)

Gisela Bengtson, Silvio Neumann, Volkan Filiz

S1. Synthesis of Monomers and Polymers

Materials: Most chemicals and solvents were delivered by Sigma Aldrich (Germany) and used as received. 5,5′,6,6′-Tetrahydroxy-3,3,3′,3′-tetramethyl-1,1′-spirobisindane (**1**) was purchased from ABCR (Germany), Maleimide-PEG-2000 (**7a**) from Specific Polymers (France) and Jeffamine®-600 from Huntsman (Germany), all used as received. 2,3,5,6-tetrafluoro-1,4-dicyanobenzene (**2**) was donated from Lanxess (Germany) and sublimed twice at 80 °C and reduced pressure. K_2CO_3 was dried at 120 °C and milled.

Synthesis within Paths 1, 2, 3, 4:

- Preparation of PIM-1 by polycondensation reaction (upper part of Scheme 1) was performed either by low temperature method following Budd and McKeown [2] and applying **1**:**2**:K_2CO_3 in molecular equivalents 1:1:2.04 (in paths 2, 3) or by high temperature method following [18, 21], applying identical amounts of the components at 150 °C and adding appropriate amounts of diethyl benzene to prevent a fast precipitation of the polymer. Reaction time is 0.5 h (PIM-1 in path 1).
- Preparation of 2,4,5-trihydroxybenzoic acid(THBA)-PEG-mono-esters (**3a-c**) and 3,4-dihydroxybenzoic acid(DHBA)-PEG-di-esters (**5a-c**) was performed by water extracting distillation of THBA resp. DHBA and PEG in boiling toluene in presence of catalytic amounts of p-toluene sulfonic acid. Reaction time 2 to 10 days, progress of reaction was followed by sampling, work up either by filtration/drying or by extraction, followed by evaporation of solvent under reduced pressure.

Monomer synthesis example 1. Preparation of **3b**: 2.000g (11.76 mmol) 2,4,5-trihydroxybenzoic acid (THBA) and 11.463 g (15.28 mmol) methoxy-PEG-750 were dissolved in toluene, 0.200 g (1.06 mmol) p-toluene sulfonic acid was added and the solution was refluxed in a Dean-Stark trap for 4 days. Progress of reaction was followed by sampling. After cooling the dark solution was poured in H_2O, organic phase separated, dried and toluene was evaporated at reduced pressure. Yield 8.62 g brown oil **3b** (80%). ^1H (DMSO-d6) δ = 3.24 (s, 3H), 3.51 (s, ca. 70H), 3.70 (m, 2H), 6.96 (s, 2H), 9.8 (br, 3H) ppm. ^{13}C (DMSO-d6) δ = 60.7 ($O\underline{C}H_3$), 63.9, 70.3 ($O\underline{C}H_2$), 109.1 (ar$\underline{C}$*H*), 119.7 (ar$\underline{C}$-*COO*), 138.9/146.0 (ar$\underline{C}$-*OH*), 166.2 (C=O) ppm. FT-IR: 3294, 2867, 1709, 1609, 1452, 1347, 1304, 1217, 1088, 1034, 947, 846 cm^{-1}.

Monomer synthesis example 2. Preparation of **5b**: 1.618 g (10.5 mmol) 3,4-dihydroxybenzoic acid (DHBA) and 4.990g (4.99 mmol) PEG-1000 were dissolved in toluene, 0.17g (0.99 mmol) p-toluene sulfonic acid was added and the solution was refluxed in a Dean-Stark trap for 4 days. Progress of reaction was followed by sampling. After cooling precipitated **5b** was filtered off, washed with toluene and dried at 50 °C/0.05 mbar. 2.5 g waxy brownish solid **5b** (yield 40%). ^1H (DMSO-d6) δ = 3.49 (s, ca. 90H), 3.72(m, 4H), 4.30 (m, 4H), 6.82 (d, 2H), 7.33 (2d, 2H), 7.39 (d, 2H), 9.5 (br, 4H) ppm. FT-IR: 3289, 2868, 1708, 1602, 1521, 1442, 1349, 1218, 1085, 946, 889, 839, 766 cm^{-1}.

- Polymers **4a-c** and **PIM(X)-*b*-PEO(Y)** multiblock copolymers were prepared by low temperature method [2].

Polymerization example 3. Preparation of **4b**: 1st step (polycondensation to PIM-1): 1.021 g (3 mmol) **1**, 0.600 g (3.0 mmol) **2** and 0.846 g (6.12 mmol) K_2CO_3 were dissolved in DMF, heated to 55 °C for 3 h. 2nd step (quenching polycondensation of PIM-1with **3b**): 0.012 (0.086 mmol) K_2CO_3 was added to the warm suspension of 1st step and stirred for 5 min, 0.0055 g (0.03 mmol) **2** was added and stirred for another 15 min, 0.059 g (0.064 mmol) **3b**, dissolved in 1 mL DMF, was added and stirred for additional 30 min.

Final suspension was poured in H_2O, filtered and dried; re-precipitation from $CHCl_3$ solution in MeOH, drying in vacuo. 1.377 g yellow powder **4b** (95% yield). SEC (MALS): M_w 52000 g/mol, M_n 40500 g/mol. ^{1}H ($CDCl_3$) δ = 1.31/1.37 (PIM-1), 2.17/2.33 (PIM-1), 3.65 (PEO), 6.42/6.81 (PIM-1). FT-IR: 2956, 2864, 2240, 1445, 1310, 1288, 1262, 1211, 1108, 1009, 874, 735 cm-1.

Polymerization example 4. Preparation of **PIM(10)-*b*-PEO(1)**: 1st step (polycondensation to PIM-1): 1.02 g (3 mmol) **1**, 0.66 g (3.3 mmol) **2** and 0.846 g (6.12 mmol) K_2CO_3 were dissolved in DMF, heated to 55 °C for 3 h, yellow suspension was poured in H_2O, filtered and dried; re-precipitation from $CHCl_3$ solution in MeOH, drying in vacuo, 1.34 g bright yellow powder (97%). M_w (RI) 9400 g/mol, M_n 5000 g/mol. 2nd step (polycondensation of PIM-1with **5b**): 0.501 g (0.05 mmol) PIM-1 (from 1st step) and 0.0667 g (0.0503 mmol) **5b** were suspended resp. dissolved in DMF, 0.161 g (1.171 mmol) K_2CO_3 was added, suspension was heated to 55 °C for 7 days; progress of reaction was followed by sampling. The final suspension was poured in H_2O, filtered off and the precipitate was dried; re-precipitation from $CHCl_3$ solution in MeOH, drying in vacuo. 0.374 g greenish yellow solid **PIM(10)-*b*-PEO(1)** (40%). SEC (MALS): M_w 99000 g/mol, M_n 70000 g/mol. ^{1}H ($CDCl_3$) δ = 1.31/1.37 (PIM-1), 2.16/2.33 (PIM-1), 3.63 (PEO), 3.8/4.4 (PEG-ester), 6.42/6.81 (PIM-1), 7.04/7.66/7.78 (DHBA) ppm. ^{13}C (DMSO-d6) δ = 21.4(PIM-1), 63.9, 68.9, 70.3 (PEO), 115.7/116.8 (PIM-1), 121.0, 122.3, 125.7, 128.6/129.3 (DHBA), 145.5, 150.9, 166.1 (DHBA) ppm. FT-IR: 2954, 2865, 2241, 1722, 1445, 1309, 1287, 1262, 1211, 1108, 1008, 874, 753 cm^{-1}.

-Synthesis within Path 4

Preparation of 9,10-dibutylanthracene-2,3,6,7-tetrol (**6**) see [14,22]. After hydrolysis of methoxy to hydroxy groups by BBr_3, **6** was obtained as an off-white powder of mp. 218–220 °C.

*Preparation of monomer **8a***: Diels-Alder reaction of **6** and maleimide-PEG-2000 (**7a**) was accomplished in p-xylene, 24 h reflux, evaporation of xylene under reduced pressure. Light brown solid **8a** (95%), mp. 46 °C. ^{1}H (DMSO-d6) δ = 1.09 (t, 6H), 1.66 (m, 4H), 1.91 (m, 2H), 2.08 (m, 2H), 2.64 (m, 2H), 2.73 (m, 2H), 3.14 (s, 2H), 3.24 (s, 3H), 3.51 (br, 176H (PEG–OCH_2), 6.56 (s, 2H), 6.67 (s, 2H), 8.50 (s, 2H), 8.68 (s, 2H) ppm.
^{13}C (DMSO-d6) δ = 15.5 (butyl$\underline{C}H_3$), 23.5(3-butyl$\underline{C}H_2$), 27.0(2-butyl$\underline{C}H_2$), 36.9(4-butyl$\underline{C}H_2$), 45.3($\underline{C}$–bicyclo), 58.4(Imido–$\underline{C}$H), 66.3(N–$\underline{C}H_2$), 70.1($\underline{C}H_2$–O), 71.6($\underline{O}CH_3$), 110.3/111.3(ar$\underline{C}$–H), 134.4/136.2(ar$\underline{C}$–bicyclo), 142.4/142.8(ar$\underline{C}$–OH), 176.1(C=O).
FT-IR: 2880, 1766, 1466, 1359, 1280, 1239, 1101(s), 1059, 595, 841 cm^{-1}. Elemental analysis: Calculated: C 57.3, H 8.59, N 0.59 Found: C 56.99, H 8.70, N 0.67 (%).

*Preparation of monomer **8b***: Maleimide-methoxy(polypropylene glycol) (**7b**) with PPO chain of ca. 600 g/mol, a viscous oil, was synthesized after [21] in 2 steps from Jeffamine®-M600 polyetheramine and maleic anhydride in 84% yield. ^{1}H (DMSO-d6) δ = 1.03 (d, 27H), 3.25 (s, 3H), 3.29-3.51 (m, 31H), 6.97 (2s, 2H). FT-IR: 2974, 2871, 1710, 1455, 1375, 1094, 1014, 926, 832 cm^{-1}.
Diels-Alder reaction of **6** and maleimide-PPO-600 (**7b**) conducted in p-xylene; 4 d reflux, evaporation of xylene under reduced pressure. **8b**, dark brown oil (70%). ^{1}H (DMSO-d6) δ = 1.04 (d, 33H), 1.65 (m, 6H), 1,91 (m, 2H), 2.08 (m, 2H), 2.65 (m, 2H), 3.25 (s, 3H), 3.37 (s, 31H), 6.58 (s, 1H), 6.68 (s, 1H), 7.06 (s, 2H), 8.58 (br, 2H), 8.69 (br, 2H) ppm. ^{13}C (DMSO-d6) δ = 14.6 (butyl$\underline{C}H_3$), 17.8(PPO–$\underline{C}H_3$), 23.7(3-butyl–$\underline{C}H_2$), 27.1/28.3 (2-butyl–$\underline{C}H_2$), 45.5($\underline{C}$–bicyclo), 49.1($\underline{C}$H–N), 58.8(imido$\underline{C}$H), 68.3/72,8/75.1 (PPO–O$\underline{C}H_2$) ,76.3(PPO–O$\underline{C}H_3$), 110.4/111.4 (ar$\underline{C}$H), 134.3, 136.5, 142.4/143.0(ar$\underline{C}$–OH), 176.7(C=O). FT-IR: 3350, 2966, 2930, 2870, 1694,1451,1375,1297,1212,1089,1025 cm^{-1}.

Polymerization example 5. Preparation of **PIM1-co-8a-1**: 0.917 g (0.375 mmol) **8a**, 4.978 g (14.625 mmol) **1**, 3.001 g (15 mmol) **2** were dissolved in DMF, 4.229 g (30.6 mmol) K_2CO_3 was added at room temperature. The suspension was heated to 55°C for 3 days and worked up as in examples 3 and 4. Re-precipitation from $CHCl_3$ solution in MeOH, yield 7.644 g of yellow powder (99% based on **8a**). SEC (MALS): M_w 43000 g/mol, M_n 33800 g/mol. ^{1}H ($CDCl_3$) δ = 1.31/1.37 (PIM-1), 2.16/2.33 (PIM-1), 3.42 (PEO), 6.42/6.81 (PIM-1) ppm. FT-IR: 3054, 2865, 2240, 1445, 1310,1288,1263,1211, 1108, 1009, 874, 753 cm^{-1}.

S2 Gas Separation

Gas permeation measurements were carried out in a fixed-volume pressure-increase apparatus in the time lag mode (descriptions of the method in [5,16,17] and literature cited therein), applying the solution-diffusion model [15]. Permeate volume is 224 cm^3, membrane area 13.8 cm^2, feed pressure 200–300 torr (26.6–40 × 10^4 Pa).

Measurements in the time lag apparatus primarily give the permeability for result, calculated by the increase of pressure on the permeate side per time after reaching a "quasi" steady state. Permeability coefficients P are calculated by eq. 1 (Vp is the permeate volume, l the actual membrane thickness, A the membrane area, pf the feed pressure (constant) and pp1 resp. pp2 permeate pressures at different times). Permeability is expressed in Barrer [1 × 10^{-10} (cm^3(STP) cm $cm^{-2}s^{-1}cmHg^{-1}$)].

$$P = \frac{Vp \times l}{A \times RT \times \Delta t} \ln(\frac{pf - pp1}{pf - pp2}) \tag{1}$$

Diffusivity coefficients D are calculated by Equation (2) using the so called time lag θ, the delay of pressure increase at start of every experiment, and the square of the membrane thickness l. Diffusivity is expressed in cm^2/s.

$$D = \frac{l^2}{6 \times \theta} \tag{2}$$

The measured time lag θ of a PIM-1 membrane (thickness 80 μm, 30 °C) varies from ca. 0.1 (He and H_2), ca. 2 (O_2), ca. 6 (CO_2 and N_2) to about 16 s for CH_4. Although the time lag and permeability measurements are repeated several times, the experimental error for small gases like H_2 and He within the highly permeable PIM-1 membrane is considerable high and the results are not really reliable.. Consequently, we concentrated on the results for the larger gas molecules under consideration being more reliable on account of their slower diffusion.

Following the solution-diffusion-model [15] permeability P is the product of diffusivity *D* and solubility *S*. Accordingly, the solubility coefficient S is calculated by eq. (3), dividing *P* by *D*.

Solubility unit is $cm^3/cm^3 \cdot cmHg$.

$$S = \frac{P}{D} \tag{3}$$
